# Sequence Variants of Toll Like Receptor 4 and Late-Onset Alzheimer's Disease

**DOI:** 10.1371/journal.pone.0050771

**Published:** 2012-12-18

**Authors:** Yen-Ching Chen, Ping-Keung Yip, Yi-Ling Huang, Yu Sun, Li-Li Wen, Yi-Min Chu, Ta-Fu Chen

**Affiliations:** 1 Institute of Epidemiology and Preventive Medicine, College of Public Health, National Taiwan University, Taipei, Taiwan; 2 Department of Public Health, College of Public Health, National Taiwan University, Taipei, Taiwan; 3 Research Center for Genes, Environment and Human Health, College of Public Health, National Taiwan University, Taipei, Taiwan; 4 Center of Neurological Medicine, Cardinal Tien Hospital, Taipei, Taiwan; 5 School of Medicine, Fu-Jen Catholic University, Taipei, Taiwan; 6 Department of Neurology, En Chu Kong Hospital, Taipei, Taiwan; 7 Department of Laboratory Medicine, En Chu Kong Hospital, Taipei, Taiwan; 8 Department of Laboratory Medicine, Cardinal Tien Hospital, Taipei, Taiwan; 9 Department of Neurology, National Taiwan University Hospital, Taipei, Taiwan; University of North Dakota, United States of America

## Abstract

**Background:**

Toll like receptor 4 (TLR4) has been related to inflammation and beta-amyloid deposition in Alzheimer's disease (AD) brain. No study has explored the association between haplotype-tagging single nucleotide polymorphisms (htSNPs) of *TLR4* and AD risk previously and *ApoE e4* status alone showed low sensitivity in identifying late-onset AD (LOAD) patients.

**Methods:**

A total of 269 LOAD patients were recruited from three hospitals in northern Taiwan (2007–2010). Controls (n = 449) were recruited from elderly health checkup and volunteers of the hospital during the same period of time. Five common (frequency≥5%) *TLR4* htSNPs were selected to assess the association between *TLR4* polymorphisms and the risk of LOAD in the Chinese ethnic population.

**Results:**

Homozygosity of *TLR4* rs1927907 was significantly associated with an increased risk of LOAD [TT vs. CC: adjusted odds ratio (AOR) = 2.45, 95% confidence interval (CI) = 1.30–4.64]. After stratification, the association increased further in *ApoE e4* non-carriers (AOR = 3.07) and in hypertensive patients (AOR = 3.60). Haplotype GACGG was associated with a decreased risk of LOAD (1 vs. 0 copies: AOR = 0.59, 95% CI = 0.36–0.96; 2 vs. 0 copies: AOR = 0.31, 95% CI = 0.14–0.67) in *ApoE e4* non-carriers. *ApoE e4* status significantly modified this association (*p*_interaction_ = 0.01). These associations remained significant after correction for multiple tests.

**Conclusions:**

Sequence variants of *TLR4* were associated with an increased risk of LOAD, especially in *ApoE e4* non-carriers and in hypertensive patients. The combination of *TLR4* rs1927907 and *ApoE e4* significantly increased the screening sensitivity in identifying LOAD patients from 0.4 to 0.7.

## Introduction

Alzheimer's Disease (AD) is the most common cause of dementia, and is characterized by accumulation of extracellular senile plaques (composed by amyloid-β peptide, Aβ), intracellular neurofibrillary tangles (containing hyperphosphorylated tau protein), and degenerating neurons [1]. In US elderlies (age 65 or older), the incidence of AD was 1.4% in 1995 and estimated to be 4.6% in 2050 [2]; it was also the sixth leading cause of death in 2009 [3]. In Taiwan elderlies, the prevalence of dementia is 1.7% to 4.3% [4]; however, AD is not the top 10 leading cause of death probably due to under-diagnosis and under-reported. As population aging quickly in most of developed countries, dementia has become an important health issue worldwide.

Beta amyloid (Aβ) load has been related to AD pathogenesis via its role in triggering the innate immune response. Toll like receptors (TLRs) recognize various pathogens infection and damaged host cells, which lead to the subsequent inflammation responses [5]. Toll-like receptor 4 (TLR4) expresses on the surface of microglia in the central nervous system and acts as the binding receptor of lipopolysaccharide (LPS) and Aβ [6], [7]. Aβ deposition increases the expression and activation of TLR4, which facilitates the uptake and clearance of Aβ in AD pathogenesis [8], [9], [10]. An animal study also showed that the Aβ load was greater in mutant *TLR4* AD mice than that of wild-type mice [11]. These reflect that *TLR4* may be an important susceptibility gene to AD via its role in innate immunity.

Two *TLR4* missense polymorphisms (Asp299Gly, equivalent to rs4986790; Thr399Ile, equivalent to rs4986791) have been related to a blunted inflammatory response to LPS [12]. Asp299Gly may affect the production of pro-inflammatory cytokines [13] and has been associated with an increased risk of AD in two Italian studies [14], [15]. However, both Thr399Ile and Asp299Gly are very rare in Chinese [16] and other populations (Indonesian, Papuan, Trio Indians in Surinam) [17]. In addition, *TLR4*/11367, a SNP in the 3′ untranslated region (UTR), has been associated with an increased risk of AD in a Chinese population [18]. In summary, few studies have assessed the association between *TLR4* polymorphisms and the risk of AD, and only 1 or 2 *TLR4* SNPs were explored previously.

TLR4 plays an important role in inflammation and age-related diseases [19]. Past studies relating *TLR4* polymorphisms to AD risk have been limited to few coding SNPs in *TLR4* (Table S1). In addition, apolipoprotein E *(ApoE) e4* is an important risk factor of AD but has low sensitivity (about 0.4) in screening late-onset (aged 65 or older) AD (LOAD) patients [20]. Therefore, this study was aimed to identify genetic marker(s) supplementary to *ApoE e4* for identifying LOAD. We used a systematic approach to select *TLR4* haplotype-tagging single nucleotide polymorphisms (htSNPs) and explored their associations with AD risk. Vascular risk factors [e.g., hypertension, hypercholesteremia, and type 2 diabetes mellitus (DM)] have been related to the pathogenesis of dementia [21], [22], [23]. This study further explored how these factors and *ApoE e4* status modify the association above.

## Materials and Methods

### Study Population

This was a case-control study. A total of 294 LOAD cases were recruited from the neurology clinics of three teaching hospitals in northern Taiwan from 2007 to 2010. Healthy controls (n = 503) were recruited from elderly health checkup and volunteers of the hospital during the same period of time. All participants were Taiwanese aged 65 years or older. Participants with the history of the following diseases were excluded: depression, Parkinson's disease, hemorrhagic or ischemic stroke, cerebral infarction, or organic brain tumors.

A questionnaire was administered to collect information on demography, vascular risk factors (hypertension, hypercholesteremia, and type 2 DM), and life style (cigarette smoking and alcohol consumption, etc.). For each participant, a blood sample was collected in a tube containing sodium EDTA. After centrifugation, genomic DNA was extracted from buffy coat by QuickGene-Mini80 system (Fujifilm, Tokyo, Japan) and then stored in a −80°C freezer. After further exclusion of participants without blood samples, a total of 269 cases and 449 controls were included for data analyses.

### Ethics Statement

The study protocol has been approved by the Institutional Review Boards of National Taiwan University Hospital, En Chu Kong Hospital, and Cardinal Tien Hospital. Written informed consent was obtained from each study participant. The consent from the legal guardian/next of kin was obtained when patients had serious cognitive impairment.

### Dementia Evaluation

At each hospital, potential dementia cases were diagnosed by a neurologist. Mini-Mental State Examination was used to evaluate their cognitive function. The diagnosis of probable dementia was evaluated by Diagnostic and Statistical Manual of Mental Disorders, Fourth Edition [24]. Head magnetic resonance imaging and computed tomography were taken to exclude participants with organic lesions. Diagnosis of AD was based on National Institute of Neurological and Communicative Disorders and Stroke and the Alzheimer's Disease and Related Disorders Association Alzheimer's Criteria [25]. The cognitive function of controls was assessed by using Short Portable Mental Status Questionnaire [26] to exclude participants with possible dementia.

### SNP Selection and Genotyping Assay

Common (frequency≥5%) *TLR4* SNPs were identified from the International HapMap Project (http://hapmap.ncbi.nlm.nih.gov) using genotype data of Han Chinese in Beijing, China (CHB). Haploview (http://www.broadinstitute.org/haploview/haploview) was used to define haplotype block by applying the modified Gabriel algorithm [27], [28]. TagSNP program [29] was used to identify htSNPs among these common SNPs.

*ApoE e4* status was determined using the assay developed by Chapman et al. [30]. Genotypes of *TLR4* htSNPs were determined by Taqman Assay (Applied Biosystems Inc., CA, USA) with genotyping success rate greater than 95% for each SNP. Quality control samples were obtained from 5% of internal samples in duplicates and genotyped together with all other samples, and the concordance rate was 100%.

### Statistical Analysis

For each SNP, the Hardy-Weinberg equilibrium (HWE) test was performed among controls to examine possible genotyping error and selection bias. Haplotype frequencies were estimated by utilizing the expectation-maximization algorithm. To control for the confounding effect of age, frequency matching was used to match cases and controls on age within an interval of 5 years. The conditional logistic regression models were performed to estimate SNP- and haplotype-specific odds ratios (OR) and 95% confidence intervals (CI) for LOAD adjusting for age, gender, education, and *ApoE e4* status. Type I error for multiple tests was controlled by Bonferroni corrections [31].

This study further explored how *ApoE e4* status and vascular risk factors (type 2 DM, hypertension, and hypercholesteremia) modified the association between *TLR4* polymorphisms and the risk of LOAD by using the likelihood ratio test. Stratified analyses were performed by these vascular risk factors to assess the association between *TLR4* polymorphisms and the risk of LOAD. SAS version 9.2 (SAS Institute, Cary, NC) was used for statistical analyses and all statistical tests were two-sided.

Statistical power for genetic main effect (SNPs and haplotypes) and gene-risk factor interaction are calculated by QUANTO (http://hydra.usc.edu/gxe/_vti_bin/shtml.dll/request.htm) and PGA (http://dceg.cancer.gov/bb/tools/pga) programs using 269 cases and 449 controls and a disease prevalence of 5%. For SNP analysis, with disease allele frequency of 26%, the power to detect a relative risk of 2.45 (codominant model with 2df) is around 0.94 (alpha = 0.05). For haplotype analysis, with disease haplotype frequency of 0.36, the power to detect a relative risk of 0.64 is about 78% (alpha = 0.05). Regarding gene-gene interaction, disease allele frequency of 0.26 and 0.23 for two SNPs, respectively, the power to detect an interaction relative risk of 3.4 is about 70% (alpha = 0.05). For gene-environment interaction, with disease allele frequency of 0.26, environmental exposure in the population of 0.48, the power to detect an interaction relative risk of 3.6 is about 95% (alpha = 0.05).

## Results

### Characteristics of Study Population

This study included 269 incident LOAD cases and 449 controls. As compared with controls, LOAD cases were older (79.8 vs. 73.2 years old); included more females (64% vs. 52%); had a lower education level (≤6 years: 51% vs. 11%), fewer with the history of hypertension (39% vs. 54%) or with hypercholesteremia (18% vs. 30%), and more *ApoE e4* carriers (39% vs. 15%, Table 1). The distributions of body mass index at age 40 s, cigarette smoking, alcohol consumption, and type 2 DM were similar between LOAD cases and controls.

**Table 1:** Characteristics of the study population.

Variables	LOAD N = 269	Control N = 449	p
Age (mean±SD)	79.8±6.3	73.2±5.8	<0.001*
Female (%)	172 (64)	234 (52)	0.002*
Education (%)			<0.001*
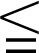 6 years	136 (51)	51 (11)	
6–12years	91 (34)	179 (40)	
>12 years	39 (15)	217 (48)	
BMI at age 40 s, kg/m^2^(mean±SD)	22.6±3.1	22.4±2.8	0.43
Cigarette smoking (%)	62 (23)	76 (17)	0.05
Alcohol consumption (%)	32 (12)	49 (11)	0.69
Type 2 DM (%)	50 (17)	63 (13)	0.11
Hypertension (%)	104 (39)	241 (54)	<0.001*
Hypercholesteremia (%)	49 (18)	133 (30)	0.001*
*ApoE e4* carriers (%)	105 (39)	66 (15)	<0.001*

* *p* value<0.05 was obtained by comparing LOAD cases and controls.

Abbreviations: LOAD, late-onset Alzheimer's disease; SD, standard deviation; BMI, body mass index; DM, diabetes mellitus; *ApoE e4*, apolipoprotein E *e*4.

### *TLR4* Polymorphisms and LOAD Risk

Five *TLR4* htSNPs (rs1927911, rs11536879, rs1927907, rs11536889, and rs7873784) were genotyped. The minor allele frequency (MAFs) of the five SNPs among controls ranged from 11% to 41%, which were similar to the MAFs of CHB genotype data from the International HapMap Project (7% to 36%, Table 2). All *TLR4* SNPs were in HWE among controls. For each SNP, the genotype frequencies were similar between cases and controls. Participants carrying two copies of variant SNP3 (rs1927907) had a significantly increased risk of LOAD [TT vs. CC: adjusted OR (AOR) = 2.45, 95% CI = 1.30–4.64, *p* = 0.004 Table 3]. This association remained significant after Bonferroni correction (α = 0.05/5). Significant association was also observed for SNP3 under the assumption of additive model (AOR = 1.36), which did not remain significant after Bonferroni correction.

**Table 2:** Characteristics of *TLR4* haplotype-tagging SNPs.

SNP name	Nucleotide change	Location	rs no.	HapMap CHB	Controls		LOAD Cases	
				MAF	MAF	HWE *p*	MAF	HWE *p*
SNP1	G→A	Intron	rs1927911	0.36	0.41	0.37	0.46	<0.01
SNP2	A→G	Intron	rs11536879	0.09	0.13	0.63	0.14	0.92
SNP3	C→T	Intron	rs1927907	0.20	0.26	0.30	0.32	<0.01
SNP4	G→C	3′ UTR	rs11536889	0.22	0.22	0.41	0.22	0.89
SNP5	G→C	3′ UTR	rs7873784	0.07	0.11	0.87	0.11	0.27

Abbreviations: UTR, untranslated region; CHB, Han Chinese in Beijing, China; HWE p, p value for Hardy–Weinberg equilibrium test; LOAD, late-onset Alzheimer's disease; MAF, minor allele frequency; SNP, single nucleotide polymorphism.

**Table 3:** Association between *TLR4* SNPs and LOAD risk.

Co-dominant model#									Additive model	
	0 copies		1 copy			2 copies				
	Case/control	AOR	Case/control	AOR (95% CI)	*p*	Case/control	AOR (95% CI)	*p*	AOR (95% CI)	*p*
SNP1	92/161	1.00	105/208	1.00 (0.65–1.54)	0.47	69/80	1.33 (0.80–2.22)	0.22	1.14 (0.88–1.47)	0.33
SNP2	196/335	1.00	61/100	1.13 (0.72–1.78)	0.21	5/9	0.43 (0.10–1.94)	0.24	0.98 (0.66–1.45)	0.90
SNP3	133/242	1.00	84/155	1.00 (0.65–1.52)	0.05	43/32	**2.45 (1.30–4.64)** *	**0.004**	**1.36 (1.03–1.80)**	**0.03**
SNP4	164/274	1.00	90/145	1.34 (0.89–2.03)	0.46	13/24	1.16 (0.45–2.97)	0.99	1.22 (0.88–1.70)	0.24
SNP5	206/341	1.00	48/86	0.97 (0.59–1.58)	0.70	5/5	0.64 (0.11–3.75)	0.63	0.93 (0.60–1.44)	0.74

All models were adjusted for age, gender, education, and *ApoE e4* status.

Abbreviations: LOAD, late-onset Alzheimer's disease; AOR, adjusted odds ratio; CI, confidence interval; SNP, single nucleotide polymorphism.

# 0 copies, wild type; 1 copy, heterozygotes; 2 copies, homozygous variants.

Numbers in bold indicates statistically significant findings (*p*<α = 0.05).

Additive model is assessing the association between number of variant allele and LOAD.

* The result remained significant (2 copies of variant SNP3, *p* = 0.004) after controlling for type I error by using Bonferroni correction (α = 0.05/5).

**Figure 1:**
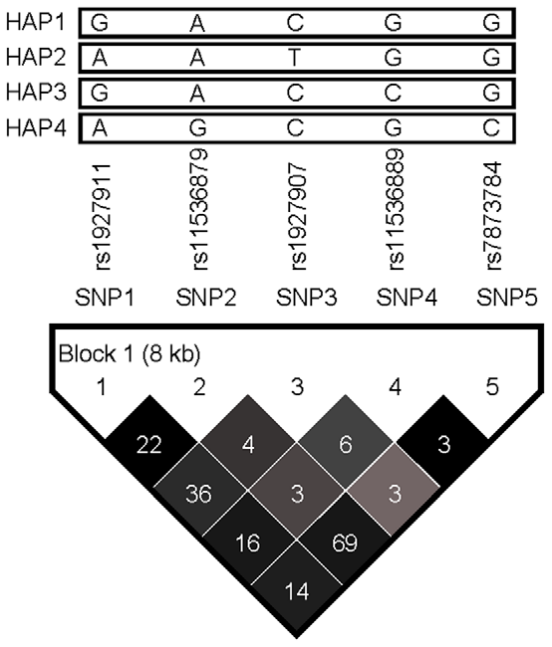
*TLR4* linkage disequilibrium (LD) plot. This plot was generated by Haploview program using data from this study. Five common (frequency≥0.05) htSNPs formed one block. The SNP name, e.g., SNP1, SNP2, etc., indicated five htSNPs genotyped in this study. Four common haplotypes were identified. The level of pairwise r^2^, which indicated the association degree between two SNPs in the LD block, was shown in the cell of the LD structure in numeric. The level of pair-wise D', which indicated the strength of LD between two SNPs, was shown in the LD structure in gray scale.

Five common (frequency≥5%) htSNPs spanning *TLR4* formed one haplotype block, which was determined by modified Gabriel et al. algorithm [27], [28] (Figure 1). Four common haplotypes with a cumulative frequency of 90% in controls were identified in *TLR4*; two of them were excluded from statistical analysis due to no controls carrying 2 copies of their corresponding haplotypes (data not shown). Participants carrying 1 copy of HAP1 (GACGG) had a significantly decreased risk of LOAD (AOR = 0.64, 95% CI = 0.42–0.97, Table 4) as compared with those carrying 0 copies of HAP1. This association did not reach statistical significance after Bonferroni correction. HAP3 was not associated with the risk of LOAD.

**Table 4:** Association between *TLR4* haplotypes and LOAD.

	Prevalence in controls, %	Co-dominant model^a^						*p*_interaction_
		0 copies		1 copy		2 copies		
		Case/Control	AOR	Case/Control	AOR (95% CI)	Case/Control	AOR (95% CI)	
HAP1 (GACGG)	36.1	134/185	1.00	102/205	**0.64 (0.42–0.97)**	33/59	0.60 (0.33–1.09)	NA
*ApoE e4* carriers								
No		83/155	1.00	64/173	**0.59 (0.36–0.96)**	16/53	**0.31 (0.14–0.67)** *	**0.01**
Yes		50/29	1.00	38/31	0.78 (0.35–1.72)	17/6	1.93 (0.54–6.82)	
Hypertension								
No		82/89	1.00	66/92	0.74 (0.41–1.32)	17/26	0.57 (0.23–1.40)	0.59
Yes		52/95	1.00	36/113	0.55 (0.29–1.04)	16/33	0.75 (0.32–1.73)	
Hypercholesteremia								
No		107/132	1.00	83/139	0.72 (0.45–1.17)	27/43	0.68 (0.34–1.34)	0.65
Yes		26/51	1.00	17/66	**0.34 (0.12–0.98)**	6/16	0.29 (0.07–1.25)	
Type 2 DM								
No		107/161	1.00	88/176	0.68 (0.43–1.08)	24/49	0.61 (0.31–1.21)	0.92
Yes		26/23	1.00	14/28	0.43 (0.13–1.38)	9/10	0.51 (0.14–1.89)	
HAP3 (GACCG)	20.3	177/290	1.00	82/136	1.36 (0.89–2.10)	10/23	1.08 (0.39–2.98)	NA
*ApoE e4* carriers								
No		102/245	1.00	56/116	**1.84 (1.11–3.06)**	5/20	1.46 (0.43–4.98)	0.11
Yes		74/44	1.00	26/19	0.70 (0.30–1.65)	5/3	0.63 (0.11–3.55)	
Hypertension								
No		108/135	1.00	52/57	1.67 (0.91–3.09)	5/15	0.95 (0.25–3.57)	0.52
Yes		69/154	1.00	30/79	1.08 (0.56–2.07)	5/8	1.17 (0.24–5.84)	
Hypercholesteremia								
No		144/202	1.00	64/94	1.27 (0.78–2.09)	9/17	1.17 (0.37–3.66)	0.87
Yes		31/86	1.00	17/42	1.91 (0.69–5.30)	1/6	1.25 (0.10–16.30)	
Type 2 DM								
No		142/246	1.00	67/120	1.29 (0.80–2.09)	10/19	1.51 (0.54–4.22)	NA
Yes		34/42	1.00	15/16	1.86 (0.60–5.82)	0/4	NA	

Abbreviations: LOAD, late-onset Alzheimer's disease; AOR, adjusted odds ratio; CI, confidence interval; DM, diabetes mellitus; HAP, haplotype; NA, not applicable; SNP, single nucleotide polymorphism; *ApoE e4*, apolipoprotein E e4.

All models were adjusted for age, gender, education, and *ApoE e4* status.

Minor alleles were underlined.

Numbers in bold indicates statistically significant findings (*p*<α = 0.05).

a 0 copies, wild type; 1 copy, heterozygotes; 2 copies, homozygous variants.

* The result remained significant (2 copies of HAP1, *p* = 0.003) after controlling for type I error by using Bonferroni correction (α = 0.05/4).

### Effect Modification by *ApoE e4* Status

*ApoE e4* carriers was associated with a significantly increased risk of LOAD (AOR = 5.05, 95% CI = 3.20–7.97) as compared with *ApoE e4* non-carriers. After stratified by *ApoE e4* status, SNP3 was significantly associated with an increased LOAD risk [TT vs. CC: AOR = 3.07, 95% CI = 1.49–6.33, *p* = 0.004, Table 5] among *ApoE e4* non-carriers, which remained statistically significant after Bonferroni correction (α = 0.05/5). However, no significant association was observed for SNP3 in *ApoE e4* carriers. Similar findings were observed for SNP4 among *ApoE e4* non-carriers (GC vs. GG: AOR = 1.82, 95% CI = 1.11–2.96, Table 5).

**Table 5:** Association between *TLR4* SNPs and LOAD risk by *ApoE e4* status.

Co-dominant model^a^							*p*_interaction_
	0 copies		1 copy		2 copies		
	Case/Control	AOR	Case/Control	AOR (95% CI)	Case/Control	AOR (95% CI)	
SNP1							
Non-carriers	56/138	1.00	63/181	1.00 (0.60–1.66)	42/62	1.50 (0.81–2.78)	0.70
Carriers	36/22	1.00	42/27	1.07 (0.47–2.45)	27/17	1.13 (0.43–2.99)	
SNP2							
Non-carriers	126/287	1.00	33/84	0.92 (0.53–1.59)	1/6	0.32 (0.03–3.10)	0.35
Carriers	70/47	1.00	28/15	1.75 (0.71–4.36)	4/3	0.75 (0.11–5.30)	
SNP3							
Non-carriers	75/210	1.00	54/127	1.24 (0.75–2.05)	29/28	**3.07 (1.49–6.33)** *	0.17
Carriers	58/31	1.00	30/27	0.54 (0.24–1.23)	13/4	1.49 (0.37–5.96)	
SNP4							
Non-carriers	94/232	1.00	61/122	**1.82 (1.11–2.96)**	7/21	1.50 (0.49–4.61)	0.06
Carriers	70/41	1.00	29/22	0.66 (0.29–1.50)	6/3	0.63 (0.11–3.53)	
SNP5							
Non-carriers	132/293	1.00	24/72	0.78 (0.43–1.44)	1/3	0.63 (0.06–7.21)	0.28
Carriers	74/47	1.00	24/13	1.63 (0.62–4.27)	4/2	0.82 (0.08–8.16)	

All models were adjusted for age, gender, and education.

Abbreviations: LOAD, late-onset Alzheimer's disease; AOR, adjusted odds ratio; CI, confidence interval; SNP, single nucleotide polymorphism; *ApoE e4*, apolipoprotein E e4.

Numbers in bold indicates statistically significant findings (*p*<α = 0.05).

a 0 copies, wild type; 1 copy, heterozygotes; 2 copies, homozygous variants.

* The result remained significant (2 copies of variant SNP3 in *AopE e4* non-carriers, *p* = 0.004) after controlling for type I error by using Bonferroni correction (α = 0.05/5).

For *TLR4* haplotypes, *ApoE e4* status significantly modified the association between *TLR4* HAP1 and the risk of LOAD (*p*_interaction_ = 0.01, Table 4). After stratified by *ApoE e4* status, *ApoE e4* non-carriers carrying 1 or 2 copies of HAP1 had a decreased risk of LOAD [1 vs. 0 copies: AOR = 0.59, 95% CI = 0.36–0.96; 2 vs. 0 copies: AOR = 0.31, 95% CI = 0.14–0.67, *p* = 0.003]. These associations remained statistically significant after Bonferroni correction (α = 0.05/4).

### Effect Modification by Vascular Risk Factors

Vascular risk factors (hypertension, type 2 DM, and hypercholesteremia) did not significantly modify the association between *TLR4* polymorphisms and the risk of LOAD. After stratification by these vascular risk factors, significant associations were observed in some subgroups as detailed below.

Hypertensive patients showed a decreased the risk of LOAD (AOR = 0.41, 95% CI = 0.28–0.61). After stratification, hypertensive patients carrying homozygosity of SNP3 had a significantly increased risk of LOAD (TT vs. CC: AOR = 3.60, 95% CI = 1.47–8.84, Table 6). After stratified by type 2 DM, non-DM patients carrying homozygosis SNP3 was associated with an increased LOAD risk (TT vs. CC: AOR = 2.34, 95% CI = 1.15–4.77, *p* = 0.002). These associations remained statistically significant after Bonferroni correction (α = 0.05/5). After stratification by hypercholesteremia, no significant association was observed (data not shown). None of the vascular risk factors significantly modified the association between *TLR4* haplotypes (HAP1 and HAP3) and the risk of LOAD; stratified analyses did not show significant association in the subgroups after Bonferroni correction (Table 4).

**Table 6:** Association between *TLR4* SNPs and LOAD risk by hypertension status.

Co-dominant model^a^							*p*_interaction_
	0 copies		1 copy		2 copies		
	Case/Control	AOR	Case/Control	AOR (95% CI)	Case/Control	AOR (95% CI)	
SNP1							
No	50/78	1.00	68/87	1.37 (0.74–2.53)	44/42	1.15 (0.55–2.38)	0.18
Yes	42/83	1.00	37/121	0.64 (0.34–1.22)	25/37	1.39 (0.65–2.98)	
SNP2							
No	117/157	1.00	40/45	1.30 (0.69–2.47)	4/4	0.31 (0.03–3.45)	0.76
Yes	79/178	1.00	21/54	0.95 (0.47–1.91)	1/5	0.47 (0.05–4.59)	
SNP3							
No	80/109	1.00	57/74	1.04 (0.58–1.86)	24/15	1.75 (0.67–4.57)	0.57
Yes	53/133	1.00	27/80	0.78 (0.40–1.54)	19/17	**3.60 (1.47–8.84)** *	
SNP4							
No	103/128	1.00	53/61	1.51 (0.84–2.72)	7/15	1.07 (0.31–3.73)	0.86
Yes	61/145	1.00	37/84	1.22 (0.66–2.26)	6/9	1.07 (0.24–4.85)	
SNP5							
No	123/159	1.00	33/40	1.17 (0.59–2.32)	3/1	0.51 (0.01–22.48)	0.59
Yes	83/182	1.00	15/45	0.73 (0.34–1.55)	2/4	0.74 (0.07–7.70)	

All models were adjusted for age, gender, education, and *ApoE e4* status.

Abbreviations: LOAD, late-onset Alzheimer's disease; AOR, adjusted odds ratio; CI, confidence interval; SNP, single nucleotide polymorphism.

Numbers in bold indicates statistically significant findings(p<α = 0.05).

a 0 copies, wild type; 1 copy, heterozygotes; 2 copies, homozygous variants.

* The result remained significant (2 copies of variant SNP3 in hypertensive persons, *p* = 0.002) after controlling for type I error by using Bonferroni correction (α = 0.05/5).

Before stratification, hypertensive patients showed a decreased the risk of LOAD (AOR = 0.41, 95% CI = 0.28–0.61).

## Discussion

**Figure 2:**
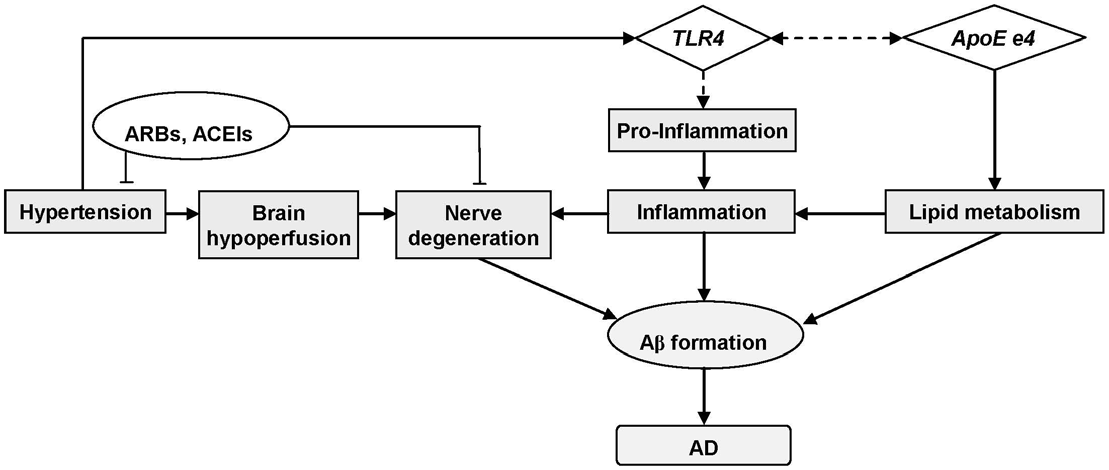
Postulated pathway of *TLR4* and factors involved in the pathogenesis of Alzheimer's disease. Solid lines indicated pathways that have been well documented; dotted lines indicated speculated pathways. Abbreviations: Aβ, beta amyloid; ARBs, angiotensin receptor blockers; ACEIs, angiotensin-converting enzyme inhibitors; *ApoE e4*, apolipoprotein E e4; AD, Alzheimer's disease.

This study is the first to assess the association between five *TLR4* htSNPs and LOAD risk. These htSNPs captured abundant genetic information in *TLR4* gene and were representative of Chinese ethnic group. We found that homozygosity rs1927907 (SNP3) was significantly associated with an increased risk of LOAD, which has not been explored previously. rs1927907 is an intronic SNP and may affect LOAD risk via regulating the alternative splicing and the subsequent protein production [32]. Sequence variants of *TLR4* may enhance the production of pro-inflammatory molecules and cytokine that leads to an increased risk of LOAD (Figure 2). Although five htSNPs are located in one linkage disequilibrium (LD) block (Figure 1), the pairwise correlations between *TLR4* htSNPs were not strong (most of pairwise r^2^<0.40). This may explain the sole significant association for rs1927907 (SNP3) but not for other *TLR4* htSNPs in the same block. Only two *TLR4* SNPs (Asp299Gly and *TLR4*/11367) were explored for AD risk in previous studies (Table S1). However, they were not included in this study because Asp299Gly is very rare in Chinese population. As for *TLR4*/11367, no sufficient information (e.g., rs number or probe/primer sequence) is available for it from the only previous study [18] or the public domain for us to perform genotyping. We further stratification our findings by two important risk factors of LOAD, gender and age, and found that homozygosity of SNP3 were significantly associated with increased risk of LOAD in females and in older people (age ≦75), respectively (Table S2 and S3). This is consistent with our understanding of LOAD that is more common in people with advancing age as the accumulation of Aβ (http://www.alz.org/alzheimers_disease_causes_risk_factors.asp) and in females due to the loss of mitochondria protection as aging [33], respectively. Previous GWASs [34], [35], [36], [37], [38] and a recent meta-analysis for GWASs of AD [39] did not identify TLR4 as a susceptibility gene for AD. This may be due to different ethnic groups explored between this study (Asian) and most previous studies (Whites). In addition, a gene with true association with AD may not be selected in the exploratory stage (stage 1) of multi-stage GWAS because of its moderate effect and a marginal p value (false negative). This phenomenon has been observed by other study [40] and may also explain why TLR4 is not in the list of susceptibility gene identified from GWASs for AD.

As for *TLR4* haplotypes, 1 copy of HAP1 was associated with a decreased risk of LOAD (Table 4). HAP1 GACGG was composed of five major alleles, which may explain the protective effect of HAP1. The association between 2 copies of HAP1 and LOAD was not significant probably due to relatively small sample size.

*ApoE e4* is a well-known risk factor of AD. This study found rs1927907 (SNP3) and HAP1 were significantly associated with an increased risk of LOAD in *ApoE e4* non-carriers and the association remained statistically significant after correction for multiple tests by using Bonferroni correction. Both previous [20] and this study observed a low sensitivity (about 0.40) by using *ApoE e4* to screen LOAD (i.e., only 40% of LOAD cases can be identified). In contrast, *TLR4* SNP3 has better sensitivity (0.51). Therefore, simultaneous testing by using both *ApoE e4* and *TLR4* SNP3 can significantly increase the screening sensitivity of LOAD to 0.7. In addition, the population attributable risk of SNP3 is even higher than that of *ApoE e4* (0.39 vs. 0.37). Therefore, *TLR4* SNP3 may be used for screening LOAD together with *ApoE e4*.

Hypertension is associated with brain-hypoperfusion and neurodegeneration, which are associated with an increased AD risk in some studies [21], [22], [23]. In contrast, some studies reported that anti-hypertension drugs (e.g., angiotensin receptor blockers or angiotensin-converting enzyme inhibitors) may provide neuroprotective effects, e.g., reduce neuronal damage and slow progression of AD [41], [42]. This may explain the protective effect of hypertension on LOAD risk in our study. However, the protective effect no longer exists when a hypertensive patient carrying homozygosity of rs1927907 (SNP3). It is possible that the protective effect from hypertensive medications is not strong enough to offset the elevated AD risk from rs1927907 (SNP3) polymorphisms and hypertension itself. Alternatively, hypertension may lead to the injury of tissue wall, which exposed the cardiovascular system to pathogens and danger signals (endogenous ligand) and then activate TLRs [43]. Elevated expression of TLR4 has been observed in AD patients and has been associated with Aβ deposition [9]. Furthermore, a recent study found that angiotensin-converting enzyme inhibitors in therapeutic dose have no effect on TLR4 expression [44]. In summary, these may explain the increased AD risk in hypertensive patients carrying variant *TLR4* via elevated inflammation responses (Figure 2).

This study had some strength. First, five *TLR4* htSNPs were identified for Chinese population to predict LOAD risk, especially among *ApoE e4* “non-carriers”. Clinically, this is helpful to identify more people with elevated risk of LOAD by simultaneously using *TLR4* SNP3 and *ApoE e4*. Second, the selection of five representative htSNPs captured abundant genetic information in *TLR4* gene (r^2^ = 0.95) as compared with the limited genetic information captured by the single *TLR4* SNP (Asp299Gly, r^2^<0.01) in two White studies [13], [14]. Importantly, the associations between *TLR4* polymorphisms (rs1927907 and HAP1 GACGG) and LOAD risk remained significant after Bonferroni correction. Therefore, these significant findings are unlikely due to chance. In addition, high false positive rate, which prevents identifying SNPs associated with the outcome because of their moderate *p* values in the exploratory stage, has been observed in genome-wide association studies [40]. This study used a systematic approach to select *TLR4* htSNPs and may thus resolve this issue. Furthermore, brain imaging helped us to exclude other diseases with similar presentation as AD.

This study had some limitations. A self-report questionnaire was used to collect information on vascular risk factors (e.g., hypertension, hypercholesteremia, and type 2 DM). Because these diseases/conditions are major health issues, participants' recall of disease/condition diagnosis and their awareness of these diseases/conditions tend to be accurate [45], [46], [47]. Therefore, information bias should not be a concern. In addition, cases were older than controls, which may lead to overestimate our findings. To solve this issue, frequency matching on age with a 5-year interval was used to compare cases with controls within the same age stratum.

This study, for the first time, found a strong association between *TLR4* polymorphisms (rs1927907 and HAP1 GACGG) and LOAD risk, especially among *ApoE e4* “non-carriers”. These associations remained significant after correction for multiple tests. The majority (about 60%) of AD patients was *ApoE e4* non-carriers and the sensitivity of *ApoE e4* has been low for LOAD. Therefore, the combination of *ApoE 4e* and *TLR4* rs1927907 can significantly increase the sensitivity from 40% (use *ApoE e4* alone) to 70%. Future large studies are warranted to explore the role of *TLR4* polymorphisms with expression data and levels of pro-inflammatory mediators on the risk of LOAD in multi-ethnic groups.

## Supporting Information

Table S1 Previous studies relating *TLR4* polymorphisms to AD risk.
(DOCX)

Table S2 Association between *TLR4* SNPs and LOAD risk by gender.
(DOCX)

Table S3 Association between *TLR4* SNPs and LOAD risk by age status.
(DOCX)
