# Supplementary material for: Sequence Variants of Toll Like Receptor 4 and Late-Onset Alzheimer's Disease
Source: PLoS One. 2012 Dec 18;7(12):e50771. doi: 10.1371/journal.pone.0050771 (PMC3525588; doi:10.1371/journal.pone.0050771)
Supplement: Table S1 — Previous studies relating TLR4 polymorphisms to AD risk. (DOCX) [file pone.0050771.s001.docx]

Table S1. Previous studies relating *TLR4* polymorphisms to AD risk

| Reference | Population, n(case:control) | Outcome | SNP (w.t./variant) | Results  OR (95% CI) | Limitations |
| --- | --- | --- | --- | --- | --- |
| Minoretti et al., 2006. | Italian,  (277:300) | LOAD | Asp299Gly* | (Asp/Gly + Gly/Gly) vs. Asp/Asp: **0.37(0.20-0.69)** | Italian only;  one SNP only. |
| Balistreri et al., 2008. | Italian,  (626:190) | AD | Asp299Gly* | Asp/Asp vs. (Asp/Gly + Asp/Gly): **1.4** (**1.03-3.52)** | Italian only;  one SNP only. |
| Wang et al., 2011. | Chinese,  (137:137) | LOAD | *TLR4*/11367 (G/C) | CC vs. GC+GG:  **8.79 (3.31-23.36)**  CC+GC vs. GG:  (**3.08**, **1.60-5.93)** | Chinese only;  one SNP only ;  small sample size. |

Abbreviations: LOAD, late-onset Alzheimer's disease; OR, odds ratio; CI, confidence interval; SNP, single nucleotide polymorphism; w.t., wild type.

*rs4986790 (A/G) is equivalent to Asp299Gly.
