# Supplementary material for: Sequence Variants of Toll Like Receptor 4 and Late-Onset Alzheimer's Disease
Source: PLoS One. 2012 Dec 18;7(12):e50771. doi: 10.1371/journal.pone.0050771 (PMC3525588; doi:10.1371/journal.pone.0050771)
Supplement: Table S2 — Association between TLR4 SNPs and LOAD risk by gender. (DOCX) [file pone.0050771.s002.docx]

Table S2. Association between *TLR4* SNPs and LOAD risk by gender

| Co-dominant model | | | | | | | | | *p*_interaction_ |
| --- | --- | --- | --- | --- | --- | --- | --- | --- | --- |
|  | 0 copies | |  | 1 copy | |  | 2 copies | |  |
|  | Case/ Control | AOR |  | Case/ Control | AOR (95% CI) |  | Case/ Control | AOR (95% CI) |  |
| SNP1 | | | | | | | | | |
| Female | 59/82 | 1.00 |  | 65/112 | 1.19 (0.63-2.23) |  | 47/40 | 1.90 (0.90-4.04) | 0.47 |
| Male | 33/79 | 1.00 |  | 40/96 | 0.92 (0.50-1.69) |  | 22/40 | 0.95 (0.44-2.02) |  |
|  | | | | | | |  |  | |
| SNP2 | | | | | | | | | |
| Female | 129/180 | 1.00 |  | 38/47 | 1.27 (0.66-2.47) |  | 3/6 | 0.48 (0.06-3.71) | 0.90 |
| Male | 67/155 | 1.00 |  | 23/53 | 0.96 (0.51-1.83) |  | 2/3 | 0.41 (0.04-4.81) |  |
|  | | | | | | |  |  | |
| SNP3 | | | | | | | | | |
| Female | 81/127 | 1.00 |  | 56/83 | 1.24 (0.68-2.27) |  | 29/14 | **4.21 (1.64-10.78)*** | 0.41 |
| Male | 52/115 | 1.00 |  | 28/72 | 0.78 (0.42-1.47) |  | 14/18 | 1.46 (0.59-3.65) |  |
|  | | | | | | |  |  | |
| SNP4 | | | | | | | | | |
| Female | 103/140 | 1.00 |  | 61/80 | 1.25 (0.69-2.25) |  | 7/11 | 0.68 (0.16-2.90) | 0.36 |
| Male | 61/134 | 1.00 |  | 29/65 | 1.45 (0.79-2.67) |  | 6/13 | 1.61 (0.49-5.31) |  |
| SNP5 | | | | | | | | | |
| Female | 134/182 | 1.00 |  | 30/40 | 0.98 (0.49-1.98) |  | 3/3 | 0.67 (0.07-6.33) | 0.94 |
| Male | 72/159 | 1.00 |  | 18/46 | 0.89 (0.45-1.79) |  | 2/2 | 0.51 (0.02-14.77) |  |

All models were adjusted for age and education.

Abbreviations: LOAD, late-onset Alzheimer's disease; AOR, adjusted odds ratio; CI, confidence interval; SNP, single nucleotide polymorphism.

***** The result remained significant after controlling for type I error by using Bonferroni correction.
