# Supplementary material for: Sequence Variants of Toll Like Receptor 4 and Late-Onset Alzheimer's Disease
Source: PLoS One. 2012 Dec 18;7(12):e50771. doi: 10.1371/journal.pone.0050771 (PMC3525588; doi:10.1371/journal.pone.0050771)
Supplement: Table S3 — Association between TLR4 SNPs and LOAD risk by age status. (DOCX) [file pone.0050771.s003.docx]

Table S3. Association between *TLR4* SNPs and LOAD risk by age status

| Co-dominant model | | | | | | | | | *p*_interaction_ |
| --- | --- | --- | --- | --- | --- | --- | --- | --- | --- |
|  | 0 copies | |  | 1 copy | |  | 2 copies | |  |
|  | Case/ Control | AOR |  | Case/ Control | AOR (95% CI) |  | Case/ Control | AOR (95% CI) |  |
| SNP1 | | | | | | | | | |
| Age < 75 years | 16/106 | 1.00 |  | 28/145 | 1.16 (0.54-2.49) |  | 12/47 | 1.64 (0.64-4.24) | 0.88 |
| Age ≥ 75 years | 76/55 | 1.00 |  | 77/63 | 0.94 (0.56-1.59) |  | 57/33 | 1.25 (0.69-2.27) |  |
|  | | | | | | |  |  | |
| SNP2 | | | | | | | | | |
| Age < 75 years | 32/230 | 1.00 |  | 21/62 | **2.26 (1.09-4.65)** |  | 0/6 | NA | NA |
| Age ≥ 75 years | 164/105 | 1.00 |  | 40/38 | 0.65 (0.37-1.13) |  | 5/3 | 0.69 (0.12-3.97) |  |
|  | | | | | | |  |  | |
| SNP3 | | | | | | | | | |
| Age < 75 years | 28/161 | 1.00 |  | 18/103 | 0.88 (0.42-1.84) |  | 6/21 | 1.94 (0.62-6.05) | 0.70 |
| Age ≥ 75 years | 105/81 | 1.00 |  | 66/52 | 1.06 (0.64-1.77) |  | 37/11 | **2.97 (1.35-6.56)*** |  |
|  | | | | | | |  |  | |
| SNP4 | | | | | | | | | |
| Age < 75 years | 34/177 | 1.00 |  | 20/100 | 1.58 (0.77-3.24) |  | 2/19 | 0.78 (0.13-4.59) | 0.71 |
| Age ≥ 75 years | 130/97 | 1.00 |  | 70/45 | 1.29 (0.78-2.11) |  | 11/5 | 1.39 (0.43-4.53) |  |
| SNP5 | | | | | | | | | |
| Age < 75 years | 35/233 | 1.00 |  | 18/52 | 1.92 (0.90-4.09) |  | 0/3 | NA | NA |
| Age ≥ 75 years | 171/108 | 1.00 |  | 30/34 | 0.55 (0.30-1.02) |  | 5/2 | 1.11 (0.14-8.70) |  |

All models were adjusted for age, gender, and education.

Abbreviations: LOAD, late-onset Alzheimer's disease; AOR, adjusted odds ratio; CI, confidence interval; NA, not applicable; SNP, single nucleotide polymorphism.

***** The result remained significant after controlling for type I error by using Bonferroni correction.
